# Navigating the Labyrinth: When the “Mesenterium Commune” Turns Colonoscopy into an Endoscopic Rollercoaster

**DOI:** 10.3390/diagnostics14111072

**Published:** 2024-05-22

**Authors:** Giacomo Emanuele Maria Rizzo, Silvia Ferriolo, Lucio Carrozza, Gabriele Rancatore, Mario Traina

**Affiliations:** 1 Endoscopy Service, Department of Diagnostic and Therapeutic Services, IRCCS-ISMETT, 90127 Palermo, Italy; 2 Department of Precision Medicine in Medical, Surgical and Critical Care (Me.Pre.C.C.), University of Palermo, 90133 Palermo, Italy

**Keywords:** Mesenterium commune, colonoscopy, virtual colonoscopy, endoscopy

## Abstract

These images involved the case of a 51-year-old woman who had a history of chronic abdominal pain, iron deficiency, and diarrhoea but no blood or mucus in her stool. She had never undergone major abdominal surgery, and her past medical evaluation diagnosed her with celiac disease, leading to the adoption of a gluten-free diet alleviating most of her gastrointestinal symptoms. However, years later, her abdominal pain returned, so she underwent an abdominal ultrasound, revealing non-specific bowel loop dilation, and a weakly positive faecal occult blood test led to a colonoscopy. Despite many efforts to advance the scope beyond the transverse colon, colonoscopy was arduous and not complete, even after several changes in decubitus and abdominal compressions. Therefore, a virtual colonoscopy was conducted, revealing no intraluminal masses, but the entire colon was located on the left side of the abdomen. Indeed, the results showed sigma and that most of the colon was curled up in the small pelvis. This rare anatomical variant, known as “Mesenterium commune” (MC), is a type of gut malrotation that develops in childhood due to a lack of omphalomesenteric loop rotation during the embryonic period. This condition can lead to episodes of intestinal obstruction, potentially resulting in an acute abdomen and leading to surgical correction. Symptoms include chronic recurring abdominal pain, nausea, vomiting, and occasionally bloody stools. Few cases of this extremely rare condition have been reported in the literature so far.

**Figure 1:**
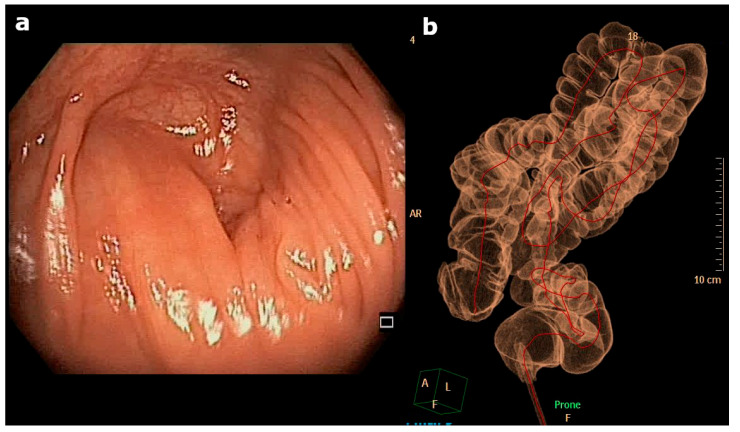
(**a**) An endoscopic view of the last endoscopic point achieved during colonoscopy: the caecum was not reached despite the vast experience of the operator, several changes in decubitus, and abdominal compressions. In this case, an anatomical alteration of the gastrointestinal tract was suspected while performing the procedure, even because of the lack of history of previous abdominal surgery and the lack of freedom in performing standard endoscopic movements. Therefore, the operator promptly and wisely interrupted the procedure, avoiding complications and permitting the performance of less-invasive examinations. (**b**) Virtual colonoscopy showing the sigma and left colon curled up in the small pelvis due to the brevity of the meso. The whole colon is located on the left side of the abdomen.

**Figure 2:**
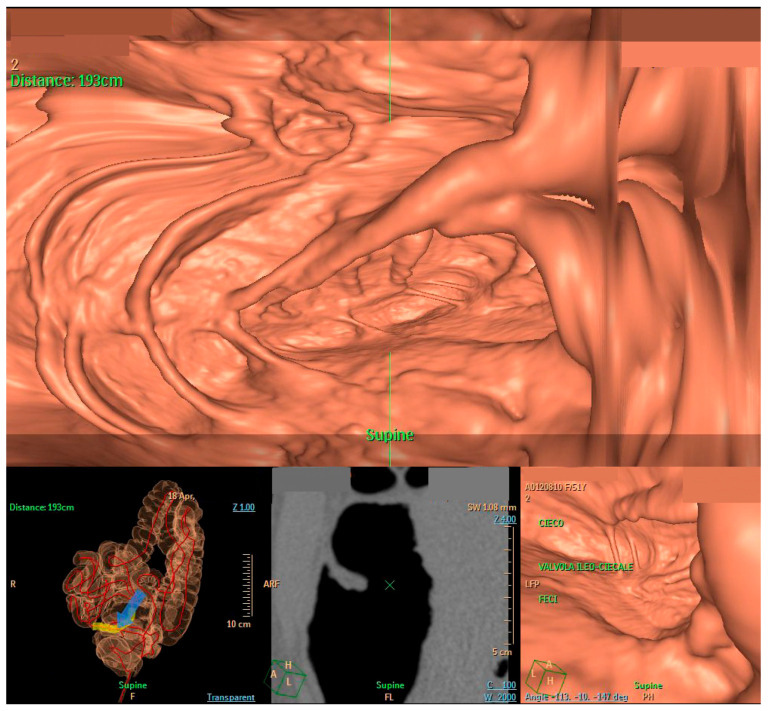
View of virtual colonoscopy, with reconstructed images of the caecum (blue arrow). The estimated distance from the anal ring was 193 cm. The patient was suffering from “Mesenterium commune” (MC), which is a rare variant of gut malrotation, developed in childhood and caused by a lack of omphalomesenteric loop rotation in the embryonic period [1]. As a result, the large bowel develops on the left side of the abdomen, and the small bowel moves and occupies the right side. This condition usually predisposes the intestinal tract to undergoing episodes of intestinal obstruction, which have been known to affect infants since the first years of the 20th century [2]. Moreover, it can present as abdominal pain or evolve into an acute abdomen, so surgical correction is sometimes the preferred therapeutic option [3]. However, rare conditions such as MC, especially in the case of an acute event or symptom recurrence, benefit from a multidisciplinary approach including at least gastroenterologists, surgeons, and radiologists. Indeed, the multidisciplinary evaluation of the best therapeutic option is fundamental for properly managing these cases when therapy is necessary.
